# Case Report: Simultaneous mucoid degeneration of the ACL and PCL in a 62-year-old man: an under-recognized diagnostic pitfall

**DOI:** 10.3389/fsurg.2025.1637907

**Published:** 2025-09-19

**Authors:** Kyun-Ho Shin, Il-Tae Jang, Seung-Beom Han

**Affiliations:** ^1^ Department of Orthopedic Surgery, Incheon Nanoori Hospital, Incheon, Republic of Korea; ^2^ Nanoori Medical Research Institute, Seoul, Republic of Korea; ^3^ Department of Orthopedic Surgery, Korea University Anam Hospital, Seoul, Republic of Korea

**Keywords:** knee, anterior cruciate ligament, posterior cruciate ligament, mucoid degeneration, arthroscopy

## Abstract

Mucoid degeneration of the cruciate ligaments is an uncommon and often under-recognized cause of chronic knee pain and motion limitation. We report the case of a 62-year-old man with an eight-year history of discomfort, progressive flexion contracture, and restricted range of motion in the right knee. Two prior arthroscopies performed elsewhere failed to identify the cause. Magnetic resonance imaging using a 1.5-Tesla scanner with 3-mm slice thickness demonstrated diffuse, increased intraligamentous signal with intact fibers in both the anterior and posterior cruciate ligaments, consistent with the “celery stalk” sign. Arthroscopy through standard anterolateral and anteromedial portals revealed hypertrophic, yellowish ligament tissue in both cruciates. Approximately 50% of the bulk of each ligament was resected with preservation of the remaining intact fibers to maintain stability. Histologic examination confirmed mucoid degeneration without inflammatory infiltration. At six months postoperatively, the patient achieved full extension, improved flexion to 130°, and complete resolution of pain, without clinical instability. This case emphasizes the importance of considering simultaneous ACL and PCL mucoid degeneration as a potential diagnosis in patients with refractory knee stiffness when common intra-articular pathologies have been excluded.

## Introduction

Chronic knee pain with restricted range of motion in middle-aged adults is most commonly attributed to meniscal pathology or degenerative joint disease (1, 2). Less commonly, mucoid degeneration of the cruciate ligaments is a rare but important differential diagnosis in cases unresponsive to conventional treatment (3–9). It is characterized by intraligamentous accumulation of mucoid material, resulting in ligament thickening with preserved fiber continuity, and typically presents with progressive stiffness and pain without instability (3–9).

Mucoid degeneration involving the anterior cruciate ligament (ACL) has been increasingly reported (3–9). Posterior cruciate ligament (PCL) involvement is uncommon (10, 11), and simultaneous degeneration of both cruciate ligaments is exceptionally rare, with only one case previously described (12). The rarity of this condition may contribute to diagnostic delay, particularly when evaluation focuses on more prevalent intra-articular pathologies.

This case report highlights that concomitant ACL and PCL mucoid degeneration may represent an under-recognized cause of refractory knee stiffness when routine work-up is negative. Diagnosis is typically established by characteristic magnetic resonance imaging (MRI) findings and confirmed through arthroscopic and histologic examination (8, 13). The prevalence of ACL mucoid degeneration has been reported at 1.8%–5.3% in MRI-based studies (14, 15). This report describes a case in a 62-year-old male with an eight-year history of discomfort and restricted active range of motion. After two inconclusive arthroscopies, the diagnosis was made by MRI, a third arthroscopic procedure, and histologic analysis. This case underscores the importance of considering this rare entity in patients with persistent symptoms despite negative findings for common intra-articular pathologies.

## Case report

A 62-year-old male presented with an 8-year history of chronic right knee pain and progressively worsening limitation of active range of motion, accompanied by episodic joint effusion. Symptoms were aggravated by prolonged standing or walking. He reported terminal extension pain, difficulty achieving deep flexion, and a subjective sensation of the knee “giving way”, without any actual episodes of mechanical instability. These symptoms significantly impaired his daily activities and quality of life.

The patient had undergone two previous arthroscopic procedures at outside institutions, 8 and 6 years earlier, respectively. The first revealed degeneration of the anterior horn of the lateral meniscus, treated with partial meniscectomy. The second involved excision of a suspected medial parapatellar plica. Despite these interventions, symptoms persisted without meaningful improvement. In both procedures, clinical suspicion focused on more common intra-articular pathologies such as meniscal lesions or synovitis, and the cruciate ligaments were not specifically evaluated for intraligamentous abnormalities.

**Figure 1:**
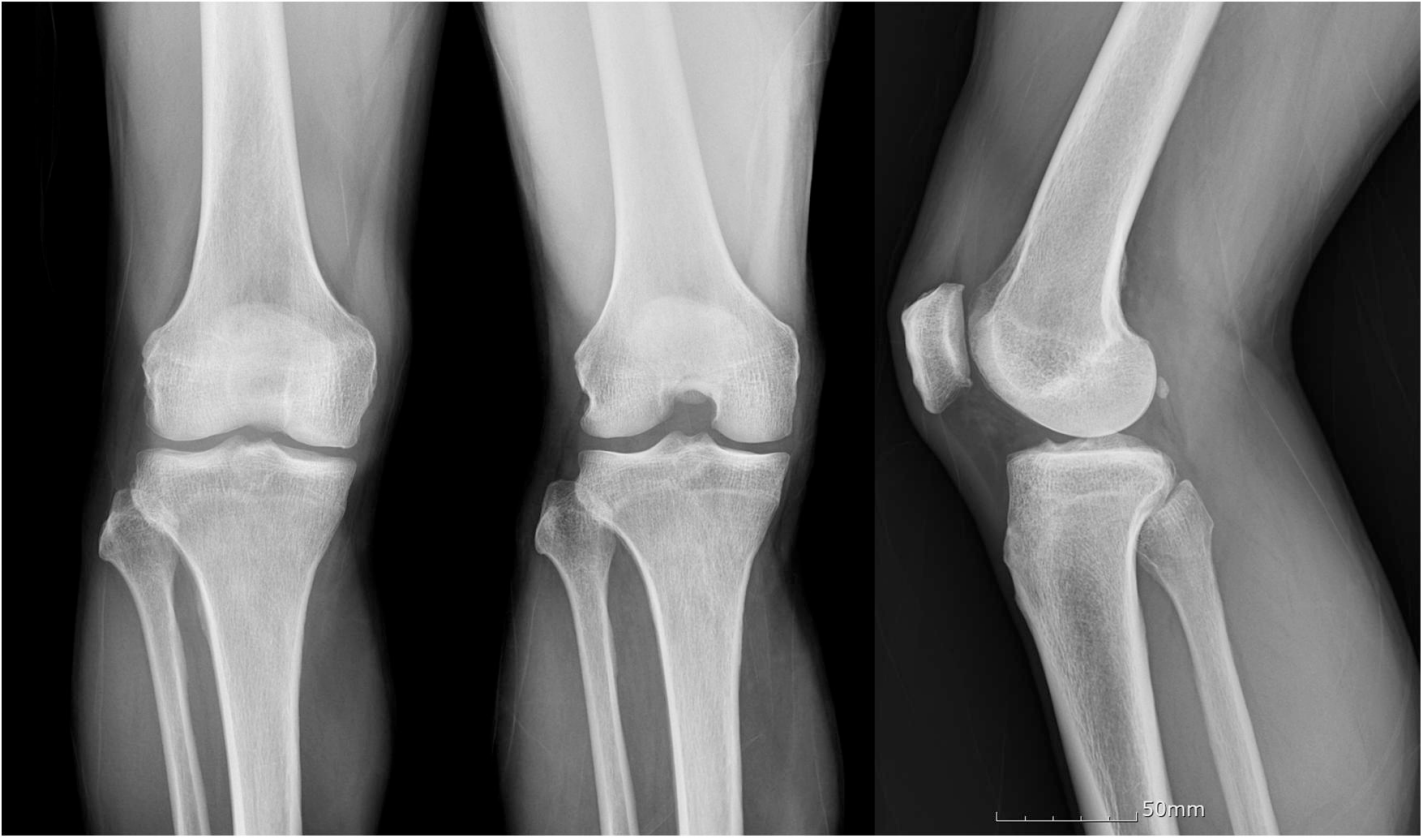
Preoperative standing anteroposterior, Rosenberg and lateral radiographs of the right knee showing mild degenerative changes and a narrowed intercondylar notch.

Physical examination at presentation revealed a 10° flexion contracture with painful limitation of further flexion beyond 100°. There was no joint line tenderness or crepitus. Lachman's, posterior drawer, and McMurray's tests were all negative. Plain standing anteroposterior, Rosenberg, and lateral radiographs showed mild degenerative changes and a narrowed intercondylar notch (Figure 1).

**Figure 2:**
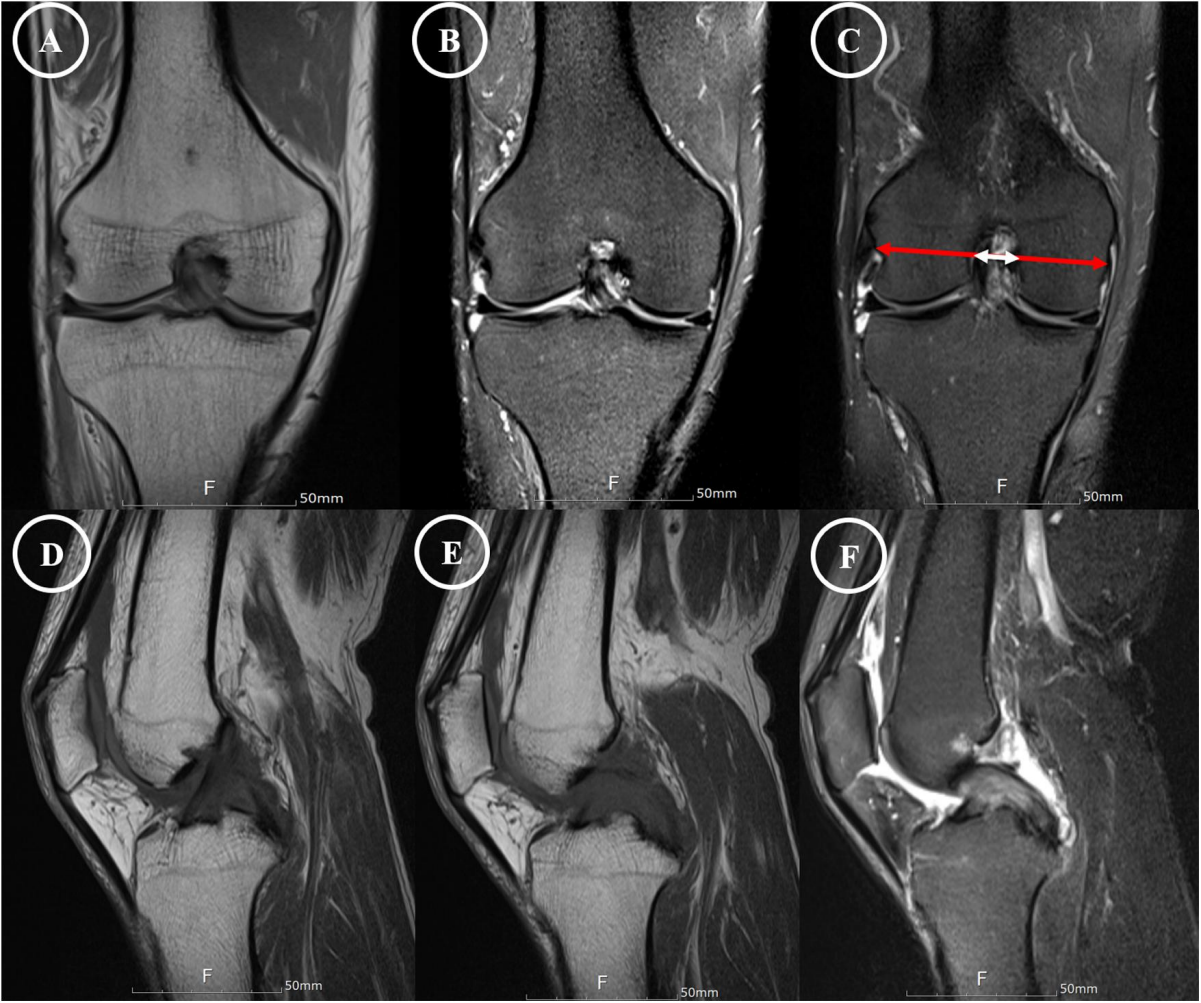
MRI demonstration of mucoid degeneration affecting both cruciate ligaments. **(A–C)** Coronal proton-density and T2-weighted images showing a markedly narrowed intercondylar notch (notch width ratio of 0.21) with close abutment between the affected ligaments. **(D–F)** Sagittal views depict the anteroposterior extent of the pathology. In both imaging planes, annotation arrows highlight the characteristic “celery stalk” sign—fusiform hypertrophy and diffuse high signal intensity within both ACL and PCL.

Magnetic resonance imaging was performed using a 1.5-Tesla scanner with 3-mm slice thickness, including T2-weighted and proton-density sequences in sagittal and coronal planes. Fusiform hypertrophy and diffuse intraligamentous high signal intensity were observed in both the ACL and PCL, with preservation of fiber continuity. The characteristic “celery stalk” appearance—linear low-signal fibers within a hyperintense background—was evident in both ligaments (Figure 2) (8, 13). The intercondylar notch was markedly narrowed, with a notch width index of 0.21 and a reverse-trapezoid configuration (Figure 2) (16, 17). No significant meniscal, chondral, or synovial abnormalities were identified.

**Figure 3:**
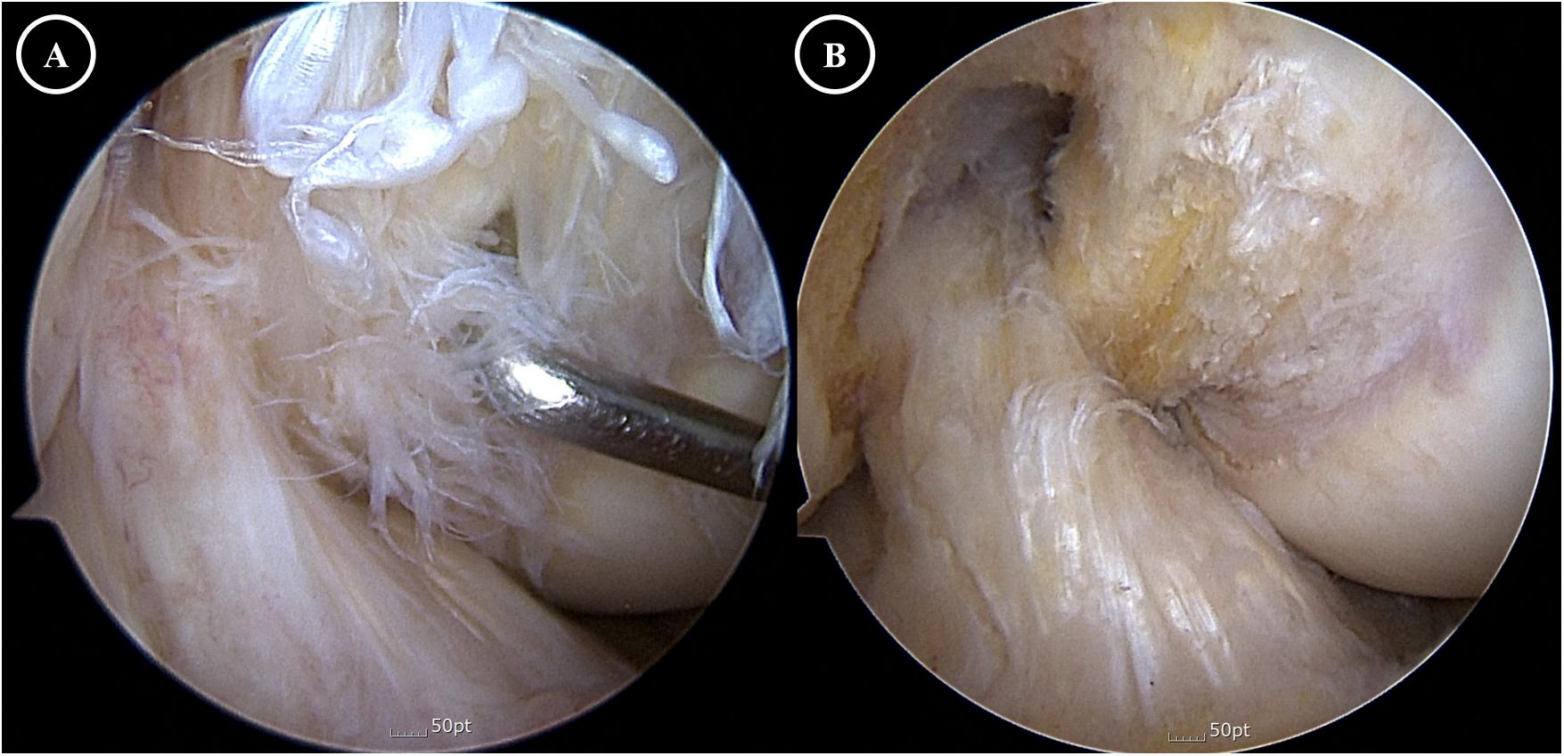
Arthroscopic findings. **(A)** Preoperative view showing a narrow intercondylar notch with hypertrophic, fraying, and degenerative ACL and PCL causing impingement. **(B)** Postoperative view after notchplasty and partial excision of mucoid tissue, revealing residual yellowish mucoid material on both ACL and PCL while demonstrating adequate notch clearance and resolution of impingement.

Arthroscopy was performed via standard anterolateral and anteromedial portals using a 30° arthroscope. The menisci and cartilage surfaces showed only mild degenerative changes. Both cruciate ligaments were markedly hypertrophied and filled with dense, yellowish fibrous material, consistent with mucoid degeneration (Figure 3). The hypertrophic ligaments occupied much of the intercondylar notch, causing mechanical impingement during passive motion. On probing, both ligaments retained normal tension and continuity. Partial excision of degenerated intraligamentous tissue was performed for both ligaments, preserving approximately 50% of the native substance. Notchplasty was also performed to widen the intercondylar space and reduce the risk of recurrent impingement (Figure 3).

Postoperatively, the patient was allowed full weight-bearing and range-of-motion exercises as tolerated from the first postoperative day. Histologic analysis of excised ligament tissue revealed faint purple mucoid material between preserved collagen fibers, consistent with intraligamentous mucoid or myxoid degeneration. Hematoxylin and eosin and Alcian blue staining demonstrated Alcian blue–positive mucinous matrix within the ligament stroma, without inflammatory infiltration or cystic architecture.

At the 6-month follow-up, the patient had complete resolution of pain and full restoration of range of motion (0°–130°). These improvements were maintained, with no recurrence of symptoms or signs of instability.

## Discussion

Mucoid degeneration is an uncommon, non-traumatic condition affecting the cruciate ligaments, characterized by intraligamentous accumulation of mucinous material resulting in fusiform thickening while maintaining structural continuity (3–8, 13). Although increasingly recognized in the anterior cruciate ligament (ACL), involvement of the posterior cruciate ligament (PCL) is far less common, and simultaneous degeneration of both cruciate ligaments is exceedingly rare (10–12). To date, only one previously published English-language report by Cho et al. has documented concurrent mucoid degeneration of both ACL and PCL, involving a patient with pain and stiffness but no instability who underwent successful partial arthroscopic excision (12). Our case differs in several respects: longer symptom duration (eight years), two prior non-diagnostic arthroscopies, and the need for notchplasty due to severe impingement confirmed on imaging and intraoperatively. In addition, Shoji et al. described a rare instance of isolated PCL mucoid degeneration, reinforcing the rarity of PCL involvement, let alone bicruciate cases (11).

Mucoid degeneration typically presents in middle-aged individuals with progressive motion restriction rather than instability, and is likely underdiagnosed. While the prevalence of symptomatic cases is unknown, MRI-based studies estimate ACL mucoid degeneration incidence at 1.8%–5.3% (14, 15), whereas clinically significant PCL or simultaneous cruciate pathology is exceedingly uncommon, appearing only in isolated case reports. Proposed mechanisms include repetitive microtrauma, degenerative changes, and synovial fluid infiltration, though the exact pathogenesis remains unclear (3–8, 13).

*n* patients with chronic knee stiffness and no identifiable intra-articular lesions, mucoid degeneration should be considered—especially when symptoms include posterior discomfort, terminal extension pain, or restricted flexion without instability. A narrow intercondylar notch with impingement between hypertrophied cruciate ligaments may underlie the limitation. Failure to suspect this condition can delay diagnosis, as in our case, where two prior arthroscopies focused on meniscal and synovial pathology, overlooking intraligamentous disease.

MRI plays a central role in diagnosis. Mucoid degeneration typically demonstrates thickened and ill-defined ligament fibers with increased intraligamentous signal, often showing the “celery stalk” sign—diffuse T2 hyperintensity with preserved fibers (8, 13). This appearance helps distinguish mucoid degeneration from cystic lesions such as intraligamentous ganglia. In our patient, both ACL and PCL exhibited classic imaging features. However, not all imaging-positive cases are symptomatic; correlation of MRI findings with clinical presentation—including range-of-motion restriction, mechanical impingement, and refractory symptoms—is essential.

Our patient's MRI showed a notch width index of 0.21, indicating substantial narrowing. A narrow notch is recognized as a risk factor for ACL injury and is often cited when deciding on notchplasty in prevention or reconstruction (16, 18). In this case, mucoid degeneration was present in both ACL and PCL. We postulate that PCL hypertrophy from degenerative changes, in combination with mucoid degeneration and a narrow notch, caused impingement between the cruciate ligaments and secondary ACL changes. This remains speculative without biomechanical or clinical evidence directly linking notch morphology to mucoid degeneration. Further research should determine whether notch shape contributes causally or only exacerbates symptoms via impingement. We performed notchplasty with partial excision to maximize three-dimensional notch volume and eliminate ACL–PCL impingement, which produced immediate symptom relief.

Arthroscopy confirmed the diagnosis. Both ligaments were hypertrophied and yellowish, filled with dense fibrous material but maintaining normal tension without rupture or cyst formation. Partial excision was performed to relieve impingement while preserving ∼50% of each ligament's volume to maintain stability. Previous arthroscopic series recommend preserving at least half of the ligament to avoid postoperative instability (6), though the key intraoperative goal is reducing tension and volume enough to abolish impingement and restore motion. Therefore, dynamic arthroscopic assessment throughout the knee's range is critical (6, 7, 9).

A limitation of this report is the short six-month follow-up, precluding assessment of long-term integrity, recurrence, or progression. Nonetheless, the immediate and sustained pain relief and motion restoration parallel outcomes in isolated ACL mucoid degeneration. __Recent evidence supports this: a systematic review by Sweed et al. of 313 knees found substantial postoperative pain relief and functional improvement after arthroscopic debridement, with symptomatic instability in only ∼6% of cases (19). Kim et al. further reported a significant association between decreased notch width index and ACL mucoid degeneration, suggesting that notch narrowing may play an anatomic role in disease pathogenesis and could warrant surgical consideration such as notchplasty (20). Although no direct studies exist on bicruciate involvement, these ACL-specific findings provide the best available evidence and support extending similar diagnostic reasoning and potential notchplasty indications to rare simultaneous ACL/PCL cases.

In conclusion, simultaneous mucoid degeneration of both ACL and PCL is an exceedingly rare but clinically significant and often under-recognized cause of chronic knee motion limitation. MRI is central to diagnosis but must be interpreted in context. Arthroscopic partial excision, with or without notchplasty, offers a safe and effective treatment for symptomatic cases.

## Key learning points

1. Bilateral cruciate involvement is exceedingly rare.
2. MRI “celery stalk” sign combined with a narrowed intercondylar notch strongly suggests the diagnosis.
3. Arthroscopic partial resection with notchplasty can provide immediate symptom relief while preserving stability.

## Data Availability

The original contributions presented in the study are included in the article/Supplementary Material, further inquiries can be directed to the corresponding author.
